# Dissection of the Genetic Basis of Maize Plant Architecture and Candidate Gene Mining Based on the MAGIC Population

**DOI:** 10.3390/genes17040399

**Published:** 2026-03-31

**Authors:** Xiaoming Xu, Kang Zhao, Yukang Zeng, Shaohang Lin, Nadeem Muhammad, Wenhui Gao, Jiaojiao Ren, Penghao Wu

**Affiliations:** 1 College of Agriculture, Xinjiang Agricultural University, 311 Nongda East Road, Urumqi 830052, China; 18690291676@163.com (X.X.); zhaokang07@yeah.net (K.Z.);; 2 Institute of Cotton Research of Chinese Academy of Agricultural Sciences, Anyang 455000, China

**Keywords:** maize, plant architecture, MAGIC population, GWAS, regulatory network

## Abstract

Background/Objectives: Plant architecture is a critical determinant of high-density tolerance and yield potential in maize (*Zea mays* L.), yet the genetic networks orchestrating these complex traits require deeper elucidation. Methods: In this study, we utilized a Multi-parent Advanced Generation Inter-cross (MAGIC) population comprising 935 recombinant inbred lines (RILs) derived from 16 diverse elite founders. A comprehensive phenotypic characterization of six pivotal architectural traits—plant height (PH), ear height (EH), ear leaf length (LL), ear leaf width (LW), tassel main axis length (TL), and tassel branch number (TBN)—was conducted across three distinct agro-ecological environments. Results: Phenotypic analysis revealed substantial natural variation and high broad-sense heritability (*H*^2^ ranging from 60% to 86%), with TBN exhibiting the most pronounced variability. Correlation architecture demonstrated a strong coupling between vertical growth traits (PH and EH, r = 0.73), while lateral leaf expansion (LW) and tassel complexity (TBN) showed significant genetic independence. Using a mixed linear model (MLM) for genome-wide association studies (GWAS), we identified 21 significant SNP–trait associations, including distinct chromosomal clusters on chromosome 8 for EH and chromosome 7 for TBN. By integrating genomic intervals with tissue-specific expression profiling, 23 core candidate genes were prioritized. Notably, *Zm00001d042528* (*FAS1*), involved in chromatin assembly, was implicated in modulating meristematic cell division for plant stature. Other key regulators included *Zm00001d020537* (*O5*) and *Zm00001d025360* (F-box protein), which were associated with reproductive organ development and leaf elongation, respectively. Conclusions: These results indicate that maize plant architecture is regulated by a modular genetic framework, with specific loci independently regulating canopy structure and source–sink components. It should be noted that the findings of this study are based solely on statistical models identifying significant associations between genetic loci and phenotypes; the biological regulatory functions of the candidate genes have not yet been experimentally validated. Nevertheless, this study provides new insights into the molecular mechanisms underlying maize morphogenesis and lays a solid theoretical foundation for molecular design breeding aimed at developing high-yielding varieties tolerant of high planting densities.

## 1. Introduction

Maize stands as the world’s preeminent cereal crop, serving as a cornerstone of global food security and livestock production systems [1]. Amid escalating global population pressures and diminishing arable land resources, enhancing grain yield per unit area has emerged as the paramount objective in maize breeding programs [2]. Contemporary high-yield breeding strategies have unequivocally demonstrated that increasing planting density represents one of the most efficacious avenues for achieving breakthrough productivity gains [3]. Nevertheless, elevated-density cultivation intensifies intraspecific competition for light, nutrients, and water; suboptimal plant architecture exacerbates susceptibility to lodging, barrenness, and disease incidence [4]. Consequently, the genetic optimization of “ideal plant architecture” has ascended to the forefront of modern maize improvement efforts [5].

Plant architecture in maize constitutes a complex, multi-component trait encompassing plant height (PH), ear height (EH), leaf morphology, and tassel branching characteristics, all of which collectively govern canopy ventilation, light penetration, and photosynthetic efficiency at the population level [6]. Specifically, PH and EH are intrinsically linked to lodging resistance; a modest reduction in EH substantially lowers the center of gravity, thereby enhancing stalk mechanical strength [7,8]. Ear leaf length (LL) and width (LW) determine leaf area index and leaf angle distribution, with compact, upright leaf orientation facilitating improved light distribution within the lower and middle canopy strata and maximizing population-level radiation use efficiency [8,9,10]. Tassel main axis length (TL) and primary branch number (TBN) critically influence source–sink resource allocation; excessive tassel size not only diverts photosynthate away from grain filling but also generates shading effects that suppress photosynthesis in upper canopy leaves, rendering tassel miniaturization an increasingly favored target in high-yield breeding programs [11,12]. Collectively, elucidating the genetic architecture underlying these six pivotal architectural traits holds profound theoretical significance and substantial practical value for the development of high-density-tolerant, high-yielding maize cultivars.

Over recent decades, genetic dissection of maize plant architecture has relied predominantly on biparental linkage mapping [13,14,15] and genome-wide association studies (GWAS) in natural populations [16,17,18]. While biparental populations enable detection of rare alleles, their limited recombination events constrain mapping resolution. Conversely, GWASs in natural populations, despite offering high resolution, remain susceptible to spurious associations arising from population structure and cryptic relatedness, and lack statistical power to identify low-frequency favorable alleles [19,20,21]. To circumvent these inherent limitations, multi-parent advanced generation inter-cross (MAGIC) populations have emerged as a transformative resource. By amalgamating extensive genetic diversity from multiple founders and dissolving linkage disequilibrium through successive generations of intermating, MAGIC populations combine exceptionally high recombination rates and mapping precision with the elimination of confounding population structure effects. Consequently, they have evolved into a formidable instrument for unraveling the genetic architecture of complex quantitative traits [22,23].

In this study, we established a maize MAGIC population capturing extensive genetic diversity and conducted comprehensive phenotypic characterization of six pivotal architectural traits: PH, EH, LL, LW, TL, and TBN. Through a GWAS, we identified the underlying genetic loci and prioritized candidate genes governing these traits. Our findings furnish novel insights into the molecular networks orchestrating maize plant architecture development and provide both genetic resources and theoretical foundations for molecular design breeding of high-density-tolerant ideotypes.

## 2. Materials and Methods

### 2.1. Experimental Materials and Population Construction

**Figure 1:**
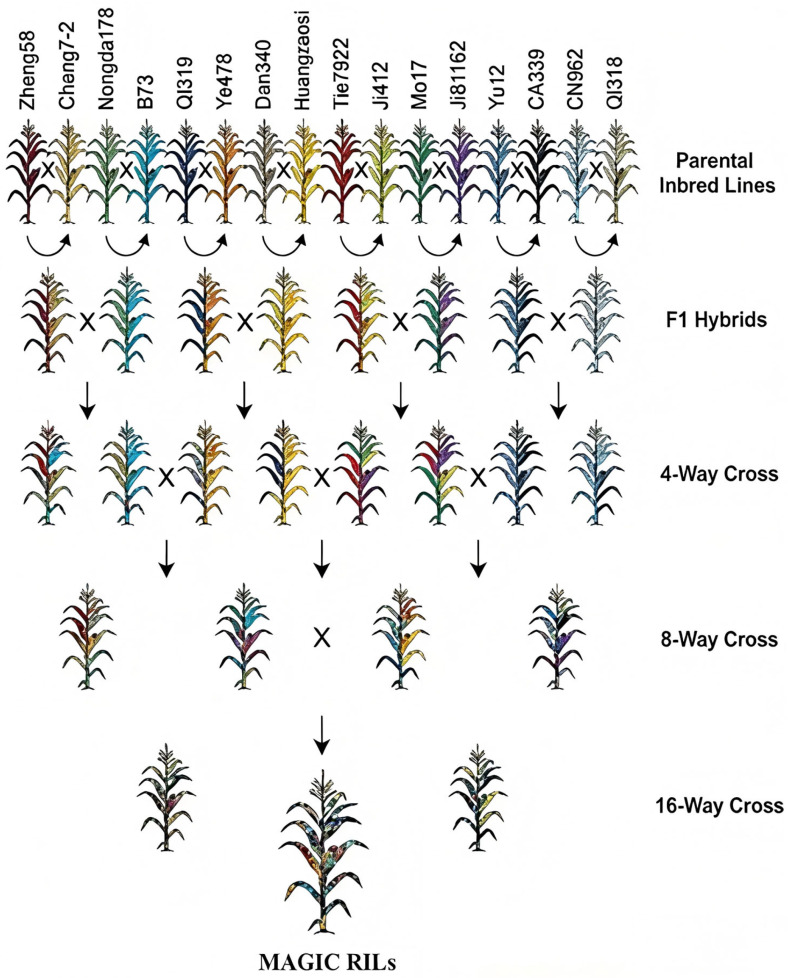
Genetic background of the MAGIC population. “
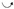
” represents the male and female parents in the cross. “↓” represents the cross-generation.

We utilized a MAGIC population constructed from 16 elite maize inbred lines with well-defined, genetically diverse backgrounds (Figure 1). The founder set encompasses Zheng 58, Chang 7-2, Nongda 178, B73, Qi 319, Ye 478, Dan 340, Huangzao 4, 7922, Ji 412, Mo 17, Ji 81162, Yu 12, CA339, CN962, and Qi 318. Population development followed a funnel hybridization scheme: initial pairwise crosses among the 16 founders generated biparental hybrids, which were subsequently advanced through four-parent and eight-parent intercrossing generations, culminating in extensive intermating and self-pollination (≥6 generations). This strategy yielded a MAGIC population comprising 935 recombinant inbred lines (RILs).

### 2.2. Field Experiment Design

Field trials were conducted across three distinct agro-ecological zones in Xinjiang from 2021 to 2023: Hutubi County (designated PX21) in 2021, Sangong Town, Changji City (PD22) in 2022, and Qitai County (PQ23) in 2023. The experimental design employed a randomized complete block design (RCBD) with two replications per environment. Each genotype was planted in single rows of 2.5 m length, with 0.25 m inter-plant spacing and 0.75 m inter-row spacing, resulting in a planting density of approximately 53,300 plants/ha.

To ensure optimal and uniform growth, high-standard agronomic management was strictly implemented at all sites. Soil preparation involved deep plowing (30 cm) followed by precise leveling. All plots utilized sub-membrane drip irrigation throughout the growing season, with total irrigation volumes maintained between 4500 and 5200 m^3^/ha to prevent drought stress. Fertilization was standardized with a basal application of diammonium phosphate (225 kg/ha) and potassium sulfate (75 kg/ha). Nitrogen (urea, 450 kg/ha total) was administered via fertigation, with 20% applied at the V6 stage, 50% at the VT stage, and 30% during the grain-filling period. Throughout the developmental cycle, all genotypes exhibited vigorous and uniform growth, with no significant biotic or abiotic interference observed.

### 2.3. Phenotypic Data Collection

Following anthesis completion in each environment, five representative plants exhibiting uniform vigor were selected per row for phenotypic assessment. The following six key architectural traits were measured:

(1) Plant height (PH): vertical distance from ground level to the tassel apex (cm).
(2) Ear height (EH): vertical distance from ground level to the node bearing the primary ear (cm).
(3) Ear leaf length (LL): distance from the leaf base to the apex of the ear leaf (cm).
(4) Ear leaf width (LW): maximum width across the ear leaf blade (cm).
(5) Tassel length (TL): distance from the tassel base to the main axis apex (cm).
(6) Tassel branch number (TBN): count of primary branches emerging from the tassel main axis base.

### 2.4. Statistical Analysis of Phenotypic Data

Raw data curation was performed in Microsoft Excel 2020, followed by descriptive statistical analysis using IBM SPSS Statistics 26. To mitigate environmental error variance, best linear unbiased predictions (BLUPs) for each trait were computed via mixed linear models implemented in META-R software (A script version based on R 4.2.0 or higher).

### 2.5. Genotyping and Quality Control

Genomic DNA was extracted from young maize leaves at the two-leaf stage using an improved CTAB method. Briefly, leaf tissues were snap-frozen in liquid nitrogen and pulverized into a fine powder. The samples were incubated with pre-heated CTAB buffer (supplemented with 1% β-mercaptoethanol) at 65 °C for 1 h, followed by extraction with chloroform–isoamyl alcohol (24:1, *v*/*v*). DNA was precipitated with pre-cooled isopropanol at −20 °C and washed twice with 75% ethanol. The resulting DNA was dissolved in double-distilled water, and its concentration and purity were quantified using a Thermo NanoDrop 2000 (Thermo Fisher Scientific, Waltham, MA, USA) spectrophotometer, while its integrity was verified by 1.0% agarose gel electrophoresis.

Genotyping was subsequently performed with a 48K liquid-phase probe capture array (48K SNP chip) across all 935 MAGIC lines, and this work was completed by Beijing Zhongyu Jinbiao Biotechnology Co., Ltd. Rigorous quality control of genotype data was conducted in PLINK v1.9, applying thresholds of minor allele frequency (MAF) ≥ 0.05 and missing rate ≤ 0.20. This procedure yielded 159,008 high-quality SNPs for downstream analyses.

Genomic DNA was extracted using the CTAB method and subsequently genotyped with a 48K liquid-phase probe capture array (48K SNP chip) across all 935 MAGIC lines. Following quality assessment and filtering of raw sequencing reads, SNP variant calling was executed through the Genome Analysis Toolkit (GATK) pipeline. Rigorous quality control of genotype data was conducted in PLINK v1.9, applying thresholds of minor allele frequency (MAF) ≥ 0.05 and missing rate ≤ 0.20. This procedure yielded 159,008 high-quality SNPs for downstream analyses.

### 2.6. Genome-Wide Association Study

To account for environmental heteroscedasticity across the three distinct agro-ecological sites, phenotypic Best Linear Unbiased Prediction (BLUP) values were estimated for each trait using a linear mixed model (LMM) in the R package lme4. The model was defined as: Y*_ijk_* = *μ* + G*_i_* + E*_j_* + R_*k*(*j*)_ + (G × E)*_ij_* + ε*_ijk_*, where Y*_ijk_* is the observed phenotype; μ is the overall mean; G*_i_*, E*_j_*, and (G × E)*_ij_* represent the random effects of genotype, environment, and their interaction, respectively; R_*k*(*j*)_ is the random effect of replicates within environments; and ε*_ijk_* is the residual error.

The resulting BLUP values, representing the stable genetic merit of each RIL while filtering out environmental noise, served as the input for a Genome-Wide Association Study (GWAS) conducted via a Mixed Linear Model (MLM) implemented in TASSEL v5.0. This model, structured as y = X*β* + P*v* + Z*μ* + e, simultaneously accounted for population structure (Q) derived from Principal Component Analysis (PCA) and individual kinship (K) using an identity-by-state (IBS) matrix to mitigate spurious associations. While a Bonferroni correction (*p* = 6.29 × 10^−8^) was initially considered for significance testing, a more balanced threshold of *p* < 1 × 10^−5^ was ultimately adopted to identify robust quantitative trait loci (QTLs) for complex architectural traits. Based on a linkage disequilibrium (LD) analysis indicating an average decay distance of 28 kb (r^2^ = 0.1) within the MAGIC population, candidate gene identification was conducted by screening a stringent physical interval of ±10 kb flanking each lead SNP. This window was selected to leverage the rapid LD decay for high-precision mapping, typically capturing proximal promoter regions and coding sequences (CDS) of causal genes directly associated with the peak marker. Putative genes within these intervals were retrieved from MaizeGDB based on the B73 Reference Genome V4 (RefGen_V4) and prioritized through tissue-specific expression profiling to identify candidates with functional relevance to maize morphogenesis.

## 3. Results and Analysis

### 3.1. Genetic Diversity and Heritability of Plant Architecture Traits in the MAGIC Population

To accurately estimate genotypic effects while minimizing environmental errors, we computed phenotypic statistics and BLUPs for PH, EH, LL, LW, TL, and TBN across the three environments (PX21, PD22, PQ23) (Table 1).

Substantial phenotypic variation was observed for all traits across environments. Notably, TBN exhibited the highest degree of variability, with coefficients of variation (CV) ranging from 14.63% to 18.70%, indicating extensive phenotypic diversity within the population. In contrast, LL and PH displayed relatively stable variation patterns. Distribution analyses revealed that absolute values of skewness and kurtosis for all traits remained below 1 in both individual environments and BLUP-integrated models, confirming adherence to normal distributions and satisfying assumptions for subsequent quantitative genetic analyses. Furthermore, BLUP values derived from multi-environment integration exhibited consistently lower standard deviations (SD) and coefficients of variation compared to single-environment estimates (e.g., PH BLUP SD = 11.66 versus 16.83–19.23 across individual environments), demonstrating effective calibration of environmental noise and enhanced representation of true breeding values.

Analysis of variance (ANOVA) indicated that genotypic variance (σ^2^_g_) reached extremely significant levels (*p* < 0.001) for all six traits, establishing substantial genetic variation as a robust foundation for downstream genetic dissection. Genotype × environment interaction variance (σ^2^_ge_) was also highly significant (*p* < 0.001) across all traits, revealing that phenotypic expression of these architectural characteristics is governed by both genetic factors and significant G × E interactions (Table 2). Broad-sense heritability (*H*^2^) estimates ranged from 60% to 86%, with TBN exhibiting the highest heritability (0.86), followed by EH (0.80), and LL showing comparatively lower heritability (0.60). These elevated heritability values indicate predominant genetic control over these traits, relatively limited environmental influence, and high phenotypic reliability—collectively supporting the suitability of these data for GWASs.

### 3.2. Genetic Correlation of Plant Architecture Traits

**Figure 2:**
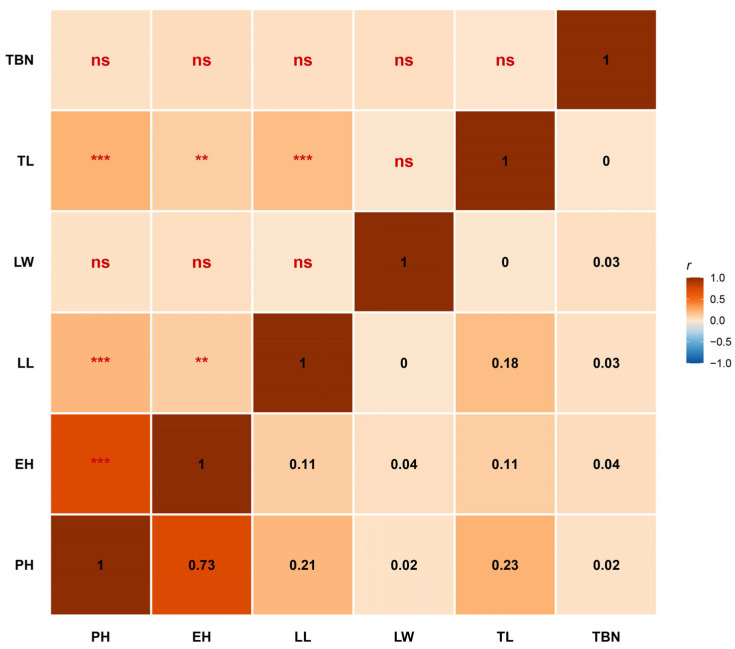
Correlation analysis of plant architecture traits in the MAGIC population. ** *p* < 0.01, *** *p* < 0.001.

Our analyses reveal that traits in this population exhibit both substantial biological interdependence and significant genetic independence, providing critical theoretical foundations for maize architectural improvement (Figure 2).

First, PH and EH displayed a highly significant positive correlation (*r* = 0.73, *p* < 0.001), reflecting tightly coordinated genetic regulation of internode elongation throughout canopy development. This pattern suggests that genes governing internode extension exert pleiotropic effects on both whole-plant and ear-zone elongation. Such tight genetic coupling implies that selection for reduced EH to enhance lodging resistance will typically concomitantly reduce overall plant stature. Second, vertical growth traits (PH, EH) showed significant positive correlations with LL and TL (*p* < 0.01), demonstrating synergistic biomass allocation between vegetative and reproductive development. Notably, however, LW and TBN exhibited exceptional genetic independence. Neither trait showed significant correlations with any other architectural character (all *p* > 0.05), indicating that lateral leaf expansion and tassel architectural complexity are governed by distinct genetic loci without physiological constraint from height development. This decoupling offers valuable breeding flexibility: LW and TBN can be manipulated independently to optimize canopy light interception and source–sink dynamics without compromising plant stature or ear position.

### 3.3. Genomic Architecture of Plant Morphological Traits via GWAS

**Figure 3:**
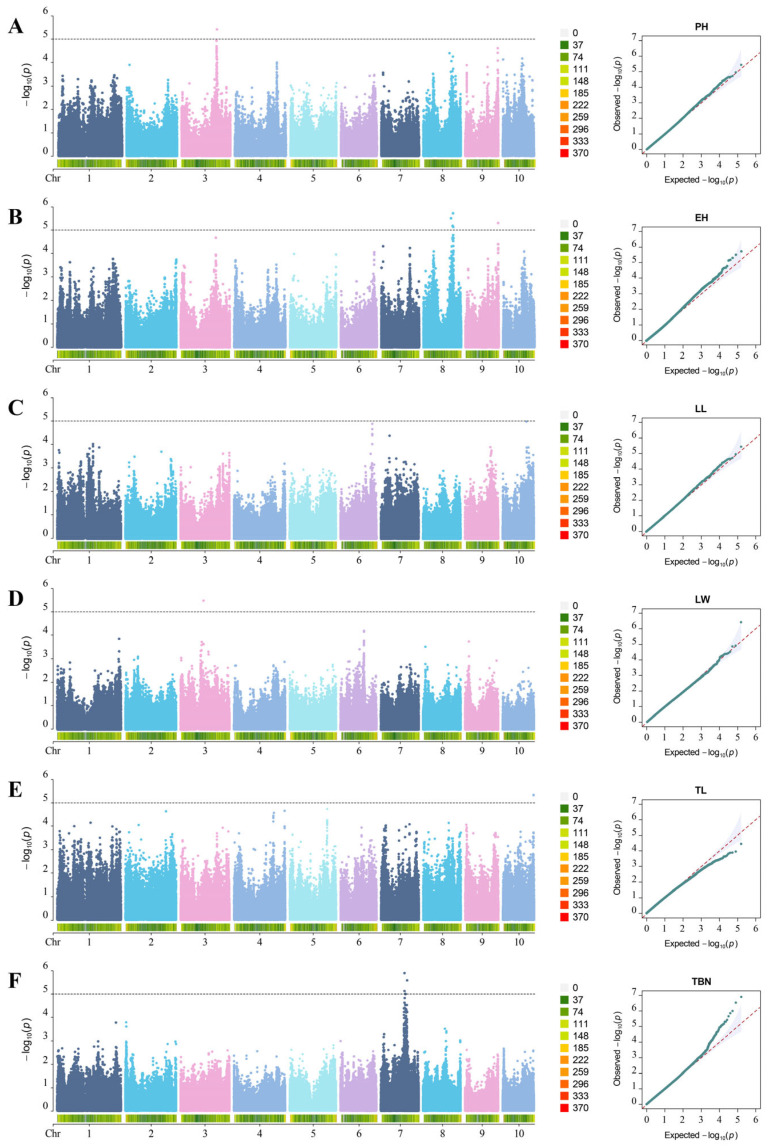
Manhattan plots of plant architecture traits in the MAGIC population. (**A**) Manhattan plot and QQ plot for PH. (**B**) Manhattan plot and QQ plot for EH. (**C**) Manhattan plot and QQ plot for LL. (**D**) Manhattan plot and QQ plot for LW. (**E**) Manhattan plot and QQ plot for TL. (**F**) Manhattan plot and QQ plot for TBN. The dashed line in Manhattan plots represents −log_10_(*p*) = 5.The dashed line in the QQ plot represents the theoretical expected value. Under the null hypothesis (i.e., no SNP is significantly associated with the phenotype), the observed values of the *p*-values should be consistent with the expected values, and the points will fall on the diagonal dashed line. If the points deviate from the dashed line and curve upwards as a whole, it indicates the presence of potential association signals or biases such as population stratification.

We conducted genome-wide association analysis using a mixed linear model (MLM) with a significance threshold of −log_10_(*p*) > 5. This approach identified 1, 5, 1, 1, 0, and 13 significant SNPs associated with PH, EH, LL, LW, TL, and TBN, respectively (Figure 3, Table 3).

PH: One significant SNP (3_170876610) on chromosome 3 (*p* = 3.67 × 10^−6^; MAF = 0.14).

EH: Five significant SNPs on chromosomes 8 and 9. The most significant association (8_142102886) resides on chromosome 8 (*p* = 3.67 × 10^−6^; MAF = 0.20).

LL: One significant SNP (10_115525664) on chromosome 10 (*p* = 9.42 × 10^−6^; MAF = 0.30).

LW: One significant SNP (3_110469000) on chromosome 3 (*p* = 3.59 × 10^−7^; MAF = 0.37).

TBN: Thirteen significant SNPs clustered on chromosome 7. The lead SNP (7_114011923) exhibited the strongest association (*p* = 1.28 × 10^−7^; MAF = 0.32). Notably, these loci displayed tight physical proximity, suggesting a potential pleiotropic or linked genetic basis for tassel architectural variation.

TL: No significant associations detected.

Quantile–quantile (QQ) plots demonstrated close concordance between observed and expected *p*-values across the majority of the distribution, confirming appropriate model calibration. Deviation from the null expectation at the extreme tail, where observed values exceeded predicted thresholds, indicates that significant SNPs represent genuine genetic effects rather than systematic bias. This upward deviation pattern is consistent with allelic variants exerting true phenotypic influence on architectural traits.

### 3.4. Haplotype Analysis and Allelic Effects

**Figure 4:**
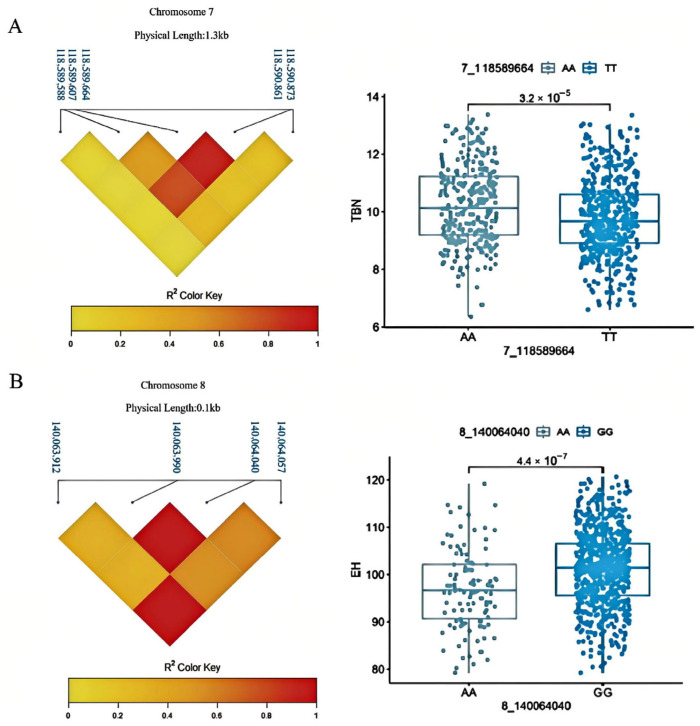
Haplotype analysis. (**A**) Haplotype analysis of EH trait 8_140064040. (**B**) Haplotype analysis of TBN trait 7_118589664.

We conducted haplotype analysis to characterize the functional allelic variants underlying significant GWAS signals. For EH, we identified a haplotype block encompassing SNP 8_140064040 on chromosome 8. Accessions homozygous for the G allele (*n* = 748) exhibited significantly greater ear height compared to those carrying the A allele (*n* = 107) (Figure 4A), indicating that the G allele confers elevated EH. For TBN, haplotype analysis of the chromosome 7 locus revealed a distinct haplotype block spanning SNP 7_118589664. The A allele was associated with significantly increased tassel branch number relative to the T allele (Figure 4B), demonstrating that this variant promotes greater tassel architectural complexity. These allelic effect estimates provide quantitative insights into the phenotypic impact of individual genetic variants, supporting their candidacy as functional polymorphisms contributing to natural variation in maize plant architecture.

### 3.5. Candidate Genes Underlying Significant Associations

**Figure 5:**
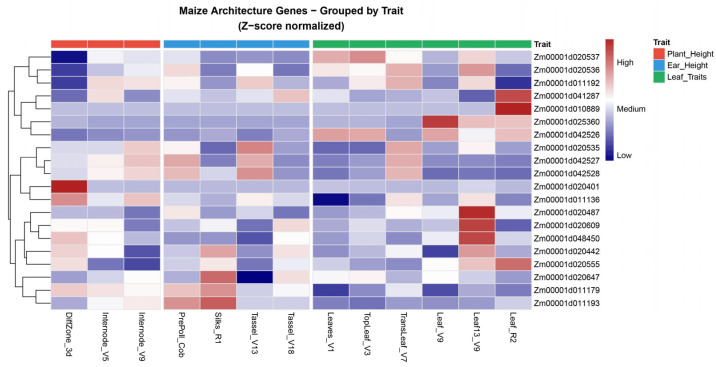
Heatmap of gene expression in various maize tissues.

We screened for candidate genes within ±10 kb (20 kb total interval) of significant SNPs based on the B73_RefGen_v4 reference genome. This analysis identified 23 primary candidates for PH, EH, LL, LW, and TBN, which we characterized through integrative analysis of tissue expression profiles (Figure 5) and functional annotations (Table 3). Eighteen genes possessed definitive functional annotations, while five encoded uncharacterized or putative proteins.

Regarding PH, the significant SNP on chromosome 3 (3_170876610) implicated three candidate genes: *Zm00001d042528*, *Zm00001d042527*, and *Zm00001d042526*. These encode chromatin assembly factor 1 subunit FAS1, a putative protein, and rhodanese/cell cycle control phosphatase, respectively. Expression heatmap analysis revealed exceptionally elevated *Zm00001d042528* transcript abundance in the differentiation zone (DiffZone_3d), suggesting potential regulation of meristematic cell division as a mechanistic basis for height variation.

Multiple significant loci were identified in EH. SNP 8_132313738 implicated *Zm00001d010889*, encoding MYB-like protein J, implicating transcriptional regulatory mechanisms. A second chromosome 8 locus (8_142607234) corresponded to *Zm00001d011192*, encoding the chloroplast carbamoyl-phosphate synthetase large subunit, connecting EH variation to chloroplast metabolic processes.

TBN exhibited pronounced gene enrichment, particularly on chromosome 7. The lead SNP (7_114011923, *p* = 1.28 × 10^−7^) pointed to *Zm00001d020433* (uncharacterized protein). Downstream of SNP 7_121383026, we identified a gene cluster comprising *Zm00001d020535* (folate-biopterin transporter), *Zm00001d020536* (aspartic protease), and *Zm00001d020537* (Opaque endosperm 5, O5). Co-expression in silks and immature tassels suggests nutrient transport and reproductive organ development as potential regulatory nodes for branch number determination. Additional candidates included *Zm00001d020555* (WAT1-related protein) and *Zm00001d020609* (riboflavin biosynthesis protein), implicating auxin-mediated cell wall development and cofactor synthesis in tassel architectural control.

For LL, the chromosome 10 SNP implicated *Zm00001d025360* (F-box protein), with expression profiling indicating active transcription during V7–V9 leaf stages—consistent with protein degradation-mediated regulation of leaf elongation. For LW, the chromosome 3 SNP (3_110469000, *p* = 3.59 × 10^−7^) corresponded to *Zm00001d041287* (uncharacterized protein), representing a novel target for lateral leaf expansion mechanisms.

## 4. Discussion

The variation in the number of significant SNPs identified across different traits reflects the inherent diversity in their genetic architectures and heritability. In our study, traits such as tassel branch number (TBN) and plant height (PH) yielded multiple robust associations, whereas no significant SNPs were detected for tassel length (TL) across all environments. This disparity can be attributed to the highly polygenic nature of TL. It is probable that TL is governed by a large number of minor-effect loci, each exerting an individual effect that falls below the stringent statistical threshold (*p* < 1.0 × 10^–5^) employed in this study. Furthermore, TL displayed a relatively low correlation with other architectural traits, indicating a more independent genetic basis. While TBN exhibited high broad-sense heritability (86%) and pronounced phenotypic variation (CV: 14.63–18.70%), making it easier for the mixed linear model to capture major-effect loci, TL appears to be more susceptible to environmental fluctuations and cumulative minor genetic variations. This suggests that TBN may be controlled by a few genomic regions with relatively large effects in this MAGIC population, whereas TL represents the “dark matter” of the maize genome that may require genomic prediction for further dissection.

Through mixed linear model analysis, we identified multiple significant SNP–trait associations that exhibit consistent effects across diverse environments. Integrating genomic screening with tissue-specific expression profiling, we prioritized 23 core candidate genes to bridge the gap between statistical association and biological morphogenesis. The candidate gene *Zm00001d042528* (*FAS1*), encoding chromatin assembly factor 1 subunit, exhibited exceptionally elevated expression in the differentiation zone (DiffZone_3d). This spatial expression pattern, coupled with the significant phenotypic variance for plant height (PH) explained by the chromosome 8 locus in our MAGIC population, suggests a mechanistic role in modulating meristematic cell division frequency through chromatin assembly dynamics. As a core component of the CAF-1 complex, *FAS1* governs diverse developmental processes [24,25,26,27,28]. The identification of FAS1 within a 16-parent recombinant background underscores its genetic stability; however, its involvement in fundamental SAM and RAM organization suggests that while it is a major determinant of plant stature, its manipulation might entail pleiotropic effects on overall vigor.

In contrast, *Zm00001d020537* (Opaque endosperm 5, O5) emerged as a more specialized candidate for tassel architecture. It exhibited co-expression in silks and immature tassels, likely participating in protein folding within the ER [29]. We hypothesize that O5 modulates nutrient allocation to reproductive organs, influencing tassel branch number (TBN) through source–sink dynamics. The high broad-sense heritabilities (up to 86%) of TBN observed in our study, combined with the tissue-specific expression of O5, designates this gene as the most promising target for ideotype breeding. Reducing TBN through O5 manipulation could minimize shading effects and optimize light interception without compromising vegetative biomass. This metabolic allocation model is further complemented by *Zm00001d020555* (WAT1-related protein), which establishes a mechanistic link between auxin transport and cell wall remodeling [4,30,31]. Together, these genes suggest that tassel branching in maize is an integrated outcome of ER-mediated metabolic status and auxin-directed biomechanical constraints.

Regarding leaf development, *Zm00001d025360* (F-box protein) exhibited active expression specifically during the V7–V9 stages. This temporal window coincides with the rapid expansion phase of middle leaves observed in our field trials. F-box proteins orchestrate organ development through the conserved “recognition–degradation” mechanism [32,33,34]. We propose that *Zm00001d025360* acts as a developmental ‘switch’ during the V7–V9 transition, specifically targeting negative regulators of cell elongation to relieve constraints on leaf expansion. Given its stage-specific activity, this gene offers a precision tool for fine-tuning leaf area index (LAI) during critical vegetative growth phases.

## 5. Conclusions

In this study, we utilized a maize MAGIC population derived from 16 diverse founders to investigate the genetic basis of six pivotal plant architecture traits across three distinct environments. Our results revealed extensive phenotypic natural variation and high broad-sense heritability (60–86%) across all traits, particularly for TBN. Through mixed linear model (MLM) analysis, we identified several significant SNP–trait associations; notably, a chromosomal cluster on chromosome 8 was found to be associated with EH, and another on chromosome 7 was linked to TBN. Based on LD decay distances and a physical interval of ±10 kb, we prioritized 23 potential candidate genes, such as *Zm00001d042528* (*FAS1*) and *Zm00001d025360* (F-box protein). It must be emphasized that while these genes are physically adjacent to significant SNPs and are involved in pathways like chromatin assembly or hormone transport based on prior literature, their specific regulatory roles in this population remain putative. This study identifies genetic correlations rather than confirmed biological mechanisms, as direct functional validation—such as through CRISPR/Cas9 or molecular assays—was not conducted within the scope of this work. Furthermore, while the significance threshold of 1 × 10^−5^ was employed to enhance the detection of QTLs for complex traits, this approach may inherently increase the risk of false-positive associations. In summary, the identified loci and candidate genes provide a preliminary genetic framework and a foundation for future research aimed at elucidating the molecular morphogenesis of maize and developing high-density-tolerant cultivars through molecular design breeding.

## Figures and Tables

**Table 1:** Phenotypic data statistics of plant architecture traits in MAGIC population.

Traits	Env	Mean	SD	CV (%)	Min	Max	Skewness	Kurtosis
PH	PX21	205.20	19.23	9.37	154.27	257.21	−0.01	−0.30
	PD22	209.48	16.83	8.03	170.55	251.11	0.18	−0.25
	PQ23	207.88	19.02	9.15	158.07	257.17	0.03	−0.21
	BLUP	208.03	11.66	5.61	175.77	237.28	−0.12	−0.30
EH	PX21	99.10	11.17	11.27	71.65	126.42	0.02	−0.34
	PD22	99.86	10.23	10.25	75.81	127.86	0.07	−0.39
	PQ23	101.44	11.75	11.58	72.95	129.35	0.11	−0.30
	BLUP	100.52	8.03	7.99	79.25	120.65	−0.02	−0.34
LL	PX21	76.55	5.97	7.80	63.81	88.51	−0.01	−0.81
	PD22	77.42	1.11	1.44	74.79	80.36	0.30	−0.27
	PQ23	74.71	5.39	7.21	63.92	87.39	0.21	−0.64
	BLUP	76.21	2.13	2.79	70.70	81.66	−0.03	−0.47
LW	PX21	8.40	0.60	7.14	6.77	9.79	−0.19	−0.30
	PD22	8.11	0.35	4.29	7.06	8.96	−0.16	−0.46
	PQ23	8.92	0.56	6.25	7.50	10.03	0.00	−0.49
	BLUP	8.49	0.38	4.48	7.45	9.44	−0.21	−0.35
TL	PX21	29.26	2.96	10.12	21.09	36.82	0.03	−0.36
	PD22	30.77	3.02	9.83	23.17	37.98	0.01	−0.49
	PQ23	29.39	2.89	9.85	22.64	36.81	0.20	−0.36
	BLUP	29.79	1.93	6.48	24.44	35.08	0.00	−0.45
TBN	PX21	9.96	1.76	17.62	5.36	14.06	0.18	−0.35
	PD22	9.91	1.45	14.63	6.31	13.69	0.21	−0.39
	PQ23	9.98	1.87	18.70	6.09	14.46	0.23	−0.55
	BLUP	10.00	1.36	13.58	6.36	13.38	0.12	−0.40

**Table 2:** Analysis of variance and broad-sense heritability for plant architecture traits in the MAGIC population.

Traits	Env	Rep	σ^2^_g_	σ^2^_e_	σ^2^_ge_	σ^2^_ε_	*H* ^2^
PH	3	2	221.04 ***	0.54	180.77 ***	89.42	0.75
EH	3	2	92.39 ***	1.23	51.49 ***	35.59	0.80
LL	3	2	9.31 ***	1.67 *	17.09 ***	3.43	0.60
LW	3	2	0.23 ***	0.17 ***	0.14 ***	0.20	0.74
TL	3	2	5.34 ***	0.64 **	4.06 ***	0.94	0.78
TBN	3	2	2.32 ***	0.00	0.89 ***	0.38	0.86

* *p* < 0.05, ** *p* < 0.01, *** *p* < 0.001.

**Table 3:** Candidate genes for plant architecture-related traits in the MAGIC population.

Trait	SNP	*p*-Value	MAF	Putative Candidate Gene_V4	Annotation of Candidate Genes
PH	3_170876610	3.59 × 10^−6^	0.14	*Zm00001d042528*	Chromatin assembly factor 1 subunit FAS1
EH	8_132313738	3.09 × 10^−6^	0.20	*Zm00001d010889*	myb-like protein J
	8_140064040	6.51 × 10^−6^	0.16	*Zm00001d011136*	NADH dehydrogenase [ubiquinone] 1 beta subcomplex subunit 2
	8_142102886	1.87 × 10^−6^	0.20	*Zm00001d011179*	uncharacterized LOC100191695
	8_142607234	7.23 × 10^−6^	0.19	*Zm00001d011193*	integral membrane protein
				*Zm00001d011192*	carbamoyl-phosphate synthase large chain, chloroplastic
	9_156640568	4.93 × 10^−6^	0.12	*Zm00001d048450*	Phosphatidylinositol-glycan biosynthesis class F protein
LL	10_115525664	9.42 × 10^−6^	0.30	*Zm00001d025360*	probable F-box protein At2g36090
LW	3_110469000	3.59 × 10^−7^	0.38	*Zm00001d041287*	uncharacterized LOC103650272
TBN	7_111464118	7.42 × 10^−6^	0.30	*Zm00001d020401*	uncharacterized LOC100273454
	7_114011848	6.91 × 10^−6^	0.18	*Zm00001d020433*	uncharacterized LOC100273454
	7_114011923	1.28 × 10^−7^	0.32	*Zm00001d020433*	uncharacterized LOC100273454
	7_114012088	1.02 × 10^−6^	0.31	*Zm00001d020433*	uncharacterized LOC100273454
	7_114012239	3.84 × 10^−6^	0.16	*Zm00001d020433*	uncharacterized LOC100273454
	7_114645301	2.23 × 10^−6^	0.36	*Zm00001d020442*	uncharacterized LOC100273454
	7_117858978	5.03 × 10^−6^	0.37	*Zm00001d020478*	Uncharacterized protein
	7_118589664	1.42 × 10^−6^	0.38	*Zm00001d020487*	ARM repeat superfamily protein
	7_121383026	9.78 × 10^−6^	0.37	*Zm00001d020535*	putative folate-biopterin transporter 6
				*Zm00001d020536*	Eukaryotic aspartyl protease family protein
				*Zm00001d020537*	opaque endosperm5
	7_121384312	8.14 × 10^−6^	0.37	*Zm00001d020535*	putative folate-biopterin transporter 6
				*Zm00001d020536*	Eukaryotic aspartyl protease family protein
				*Zm00001d020537*	opaque endosperm5
	7_122133158	8.73 × 10^−6^	0.31	*Zm00001d020555*	WAT1-related protein
	7_124931069	5.04 × 10^−6^	0.44	*Zm00001d020609*	Riboflavin synthase-like superfamily protein
	7_126453372	2.98 × 10^−7^	0.39	*Zm00001d020647*	uncharacterized LOC100191489

## Data Availability

All data generated or analysed during this study are included in this published article.

## Abbreviations

MAGIC	Multi-parent Advanced Generation Inter-cross
PH	plant height
EH	ear height
LL	ear leaf length
LW	ear leaf width
TL	tassel main axis length
TBN	tassel branch number
GWAS	Genome-wide association study
MLM	mixed linear model
PX21	Hutubi County in 2021
PD22	Sangong Town, Changji City, in 2022
PQ23	Qitai County in 2023
RCBD	randomized complete block design
BLUP	best linear unbiased predictions
DNA	Deoxyribonucleic Acid
CTAB	Cetyltrimethylammonium Bromide
SNP	single-nucleotide polymorphism
GATK	Genome Analysis Toolkit
MAF	Minor Allele Frequency
QQ	quantile–quantile plots
LD	linkage disequilibrium
CV	Coefficient of Variation
SD	standard deviation
ANOVA	Analysis of variance
SAM	shoot apical meristem
RAM	root apical meristem
ER	endoplasmic reticulum
